# Views of Community Managers on Knowledge Co-creation in Online Communities for People With Disabilities: Qualitative Study

**DOI:** 10.2196/jmir.7406

**Published:** 2017-10-10

**Authors:** Julia Amann, Sara Rubinelli

**Affiliations:** ^1^ Swiss Paraplegic Research Nottwil Switzerland; ^2^ Department of Health Sciences and Health Policy University of Lucerne and Swiss Paraplegic Research Lucerne/Nottwil Switzerland

**Keywords:** community networks, internet, patient-centered care, telemedicine, community participation, co-creation

## Abstract

**Background:**

The use of online communities to promote end user involvement and co-creation in the product and service innovation process is well documented in the marketing and management literature. Whereas online communities are widely used for health care service provision and peer-to-peer support, only little is known about how they could be integrated into the health care innovation process.

**Objective:**

The overall objective of this qualitative study was to explore community managers’ views on and experiences with knowledge co-creation in online communities for people with disabilities.

**Methods:**

A descriptive qualitative research design was used. Data were collected through semi-structured interviews with nine community managers. To complement the interview data, additional information was retrieved from the communities in the form of structural information (number of registered users, number and names of topic areas covered by the forum) and administrative information (terms and conditions and privacy statements, forum rules). Data were analyzed using thematic analysis.

**Results:**

Our results highlight two main aspects: peer-to-peer knowledge co-creation and types of collaboration with external actors. Although community managers strongly encouraged peer-to-peer knowledge co-creation, our findings indicated that these activities were not common practice in the communities under investigation. In fact, much of what related to co-creation, prototyping, and product development was still perceived to be directed by professionals and experts. Community managers described the role of their respective communities as informing this process rather than a driving force. The role of community members as advisors to researchers, health care professionals, and businesses was discussed in the context of types of collaboration with external actors. According to the community managers, most of the external inquiries related to research projects of students or health care professionals in training, who often joined a community for the sole purpose of recruiting participants for their research. Despite this unilateral form of knowledge co-creation, community managers acknowledged the mere interest of these user groups as beneficial, as long as their interest was not purely financially motivated. Being able to contribute to advancing research, improving products, and informing the planning and design of health care services were described as some of the key motivations to engage with external stakeholders.

**Conclusions:**

This paper draws attention to the currently under-investigated role of online communities as platforms for collaboration and co-creation between patients, health care professionals, researchers, and businesses. It describes community managers’ views on and experiences with knowledge co-creation and provides recommendations on how these activities can be leveraged to foster knowledge co-creation in health care. Engaging in knowledge co-creation with online health communities may ultimately help to inform the planning and design of products, services, and research activities that better meet the actual needs of those living with a disability.

## Introduction

### Online Health Communities

Online health communities (OHCs) are an increasingly popular source of health information and peer-to-peer support, particularly for patients with chronic health conditions [1-4]. They enable individuals to connect and exchange their experiences with symptoms, treatments, side effects, and strategies for self-management [5]. By publicly sharing their experiential expertise and health advice, individuals can provide important support to others in need [6-8]. Building on the findings of earlier work [9-13], this study proposes that in addition to the several purposes of OHCs, including their role as peer-support networks, these communities can also be used as platforms to promote patient participation in health care. By enabling patients to actively participate in the health care discourse online, OHCs can, in turn, also foster collaboration and knowledge co-creation between patients, researchers, health care professionals, and businesses, allowing for a multilateral exchange of knowledge and ideas to create new knowledge [14,15]. The main objective of this paper was to explore community managers’ views on knowledge co-creation taking place in existing online communities for people with disabilities.

### Patient Participation

Traditionally, patient participation (also commonly referred to as *patient involvement* or *patient engagement*) refers to the active role that patients can play in their own care process by engaging in activities such as shared decision making and self-management of their health condition [14,16]. As such, it emphasizes patients’ rights to choose and control medical decisions concerning their own health and has been recognized as a promising strategy to enhance individual care and health outcomes [17].

More recently, the role of peer-to-peer support has gained increasing attention from health care research and practice. Increasingly, patients are recognized as an important resource for other patients and as allies for health care professionals [18]. They can complement existing health care services by sharing their experiential knowledge to provide emotional and practical support to individuals facing similar health issues [19]. In this context, patients can become health coaches who guide and motivate those facing similar struggles to adopt or modify certain health behaviors, for example, to engage in effective self-management practices [20,21].

Being deeply rooted in the traditional view on patient participation, we propose an extension of the concept of patient participation to account for patients’ contributions to the planning and design of health care services, products, and research by co-creating knowledge with other patients, health care professionals, researchers, or businesses. Our conceptualization of patient participation draws on the literature on user-driven innovation in general [22] and patient-driven innovation in particular [10,23], which recognizes the patient as an active collaborator in the health care innovation process. Informed by earlier work [24], we consider the health care innovation process to be concerned with the identification and introduction of new concepts and ideas related to services, processes or products that seek to improve treatment, diagnosis, education, outreach, prevention, and research with the ultimate goal of improving health outcomes, quality, safety, efficiency, and cost-effectiveness [9]. As such, our conceptualization of patient participation also relates to work in the field of codesign and co-creation in health care quality improvement, which actively involves patients, family members, and health care professionals in the planning and design of health services [25-27]. In the context of this paper, the term patient participation thus refers to a patient’s active role in the health care process that is not limited to his or her own health but also accounts for patients’ contributions to the planning design of health care services, products, and research through knowledge co-creation.

### Knowledge Co-creation

Knowledge constitutes a key element to foster product and service innovation and has thus created much scholarly interest, particularly in the marketing and management literature. Whereas there is no universally accepted definition of co-creation, it has been described as an act of collective creativity, with applications ranging from product and service design to more abstract spheres of value creation taking place between two or more individuals who may or may not belong to the same actor group (eg, patients, researchers, and health care professionals) [28]. It builds on but extends beyond knowledge collaboration, which involves providing and receiving information or support (eg, peer-to-peer support), in that it constitutes an exchange that leads to the creation of new knowledge and ideas. As such, co-creation can, for example, involve a group of patients who by sharing their know-how and lived experience develop new strategies for managing a specific health problem, but it can also refer to the exchange taking place between patients and health care professionals who work together to develop a new health service or information brochure. Following Bagayogo and colleagues [29], we thus consider knowledge co-creation in health care to be a voluntary collaborative process that involves individuals, including patients, researchers, and health care professionals, sharing and creating new knowledge about health care services, products, and research. The creation of new knowledge, in turn, constitutes a key driver for product and service innovation [30], which is of key interest to health care institutions.

One of the main challenges health care organizations face in this context is the transfer of tacit knowledge (*know-how*, lived experience) that is stored in the minds of different actors, including patients, health care professionals, and researchers into explicit organizational knowledge [31,32]. To overcome this challenge, Kohlbacher [33] suggests that whereas knowledge creation is usually conducted in a unilateral way, where firms generate, collect, and analyze information about customers, the focus should be on knowledge co-creation. Here, he refers to Prahalad and Ramaswamy [34] who describe how “co-creation converts the market into a forum where dialogue among the consumer, the firm, consumer communities, and networks of firms can take place” to create value for the stakeholders involved in the co-creation process. Similarly, Sawhney and Prandelli [35] argue that there is a need for a shift from the perspective of the firm as sole knowledge creator to a perspective where firms are co-creators of knowledge and learn to co-create value with their consumers.

In health care, however, knowledge co-creation is still in its infancy. Although the value of patient participation is widely acknowledged in the domains of self-management and peer-to-peer support (knowledge collaboration), the patient’s role as an active participant in the health care innovation process remains contested. Similar to traditional market research, health care organizations and researchers are currently more focused on gaining information about patients rather than co-creating knowledge with patients.. Health care organizations, for example, rely on patient satisfaction indicators as a basis for improvement of their offers, rather than directly involving patients in the planning and design of health care services [36].

However, there are some noteworthy advances, particularly in the field of health care quality improvement, that draw on design-thinking principles to enable knowledge co-creation between service users and health care providers [27,37-40]. Co-creation projects have been carried out in different settings, including emergency departments, intensive care units, cancer services, and mental health services, resulting in ideas for modification of processes, practices, and clinical environments, as well as tangible service changes and impacts on patient experiences [39]. Research in the field of patient-driven innovation further shows that patients can also innovate and co-create among themselves without requiring a health care organization to initiate or mediate these activities. The Nightscout initiative constitutes an example of such a collaborative patient-driven effort. The Nightscout community has created an open-source do-it-yourself mobile technology system for individuals living with type 1 diabetes, which can be accessed, used, and modified by anyone. In doing so, it allows patients, their caregivers, and health professionals to better monitor, predict, and manage diabetes using personalized tools [41].

So while there is more and more evidence on the innovative potential of patients’ contributions to the health care innovation process, methods and ideas on how to involve them as active partners are less established [14,42]. Moreover, time and resource constraints constitute an additional challenge to co-creation in health care [38].

### Online Health Communities as Platforms for Co-creation

With their increasing interactivity, social media technologies provide an ideal platform to foster co-creation between the different stakeholders in health care [14,43]. Here, social media technologies refer to Web-based technologies that enable individuals around the globe to connect with each other to share and exchange information via virtual platforms, often also referred to as online communities or networks [44]. Technologies for these Web-based communities include, among others, online message boards (forums), chat rooms, as well as an ever-expanding landscape of social networks such as Twitter, Facebook, YouTube, or Instagram [45].

There is an extensive body of literature investigating the role of OHCs in the context of peer-to-peer support [5,46,47], with findings suggesting there is indeed an ongoing exchange taking place in OHCs, leading to the development of rich databases of experiential knowledge that individuals can draw on [21]. Particularly, people with rare and chronic health conditions are likely to seek and benefit from emotional and practical online peer-to-peer support [48,49]. Rains and colleagues [46] found that informational and emotional support messages were, in fact, the most prevalent forms of exchange in more general health–related online contexts, whereas action-facilitating forms of knowledge were more characteristic in the context of chronic health conditions. However, few attempts have been made to better understand the process of social media-enabled knowledge co-creation, where the exchange of information between individuals results in the creation of new knowledge and ideas. Here, the work of Bagayogo and colleagues [29] constitutes a noteworthy exception. The authors propose a three-stage model that explains the process of knowledge co-creation through social media. The first phase, initiation, refers to users sharing or requesting knowledge related to aspects such as diagnosis, treatment, or self-management. In the transition phase, an increasing number of users then collaboratively engage in a discussion, supplementing information that other community members may have shared, or responding to questions posed by others. In the normalization phase, the main focus is on reaching consensus on a specific idea, such as, for example, a self-management strategy [29].

More and more health care organizations are realizing the significance of OHCs as an important form of complementary service to enhance the overall quality of health care services delivery. The key focus of most of these OHCs is to provide a platform for patient support, where patients can interact with others to obtain and provide emotional support in disease management and care [50]. However, whereas many other industries have started to harness the innovative potential of these communities by using them as a venue for customer co-innovation and value co-creation, health care organizations are lagging behind [9,51]. One of the key issues health care organizations face related to deploying the so-called online patient innovation communities is the fact that they are resource-intensive endeavors that require a clear strategy and organizational support [9].

Given that building and maintaining an entirely new community from scratch requires an essential investment without guaranteed success [52], an alternative option would be to engage existing OHCs in a co-creation process. An example of this form of co-creation from the consumer goods industry is the NikeTalk community [53]. NikeTalk is an independent basketball-enthusiast community with no official affiliation with the same-named shoe manufacturer, Nike. The community is occasionally approached by Nike to gain users’ insights and ideas for new designs and features. There are even examples showing that sometimes users actively initiate contact with sporting goods companies on their own [53].

### Objectives of This Study

With this study, we seek to contribute to the literature on patient participation in health care, focusing particularly on the role that OHCs can play in fostering knowledge co-creation among the different stakeholders in health care. Here, the term patient participation, as outlined earlier, refers to a patient’s active role in the health care process that is not limited to his or her own health but also accounts for patients’ contributions to the planning design of health care services, products, and research through knowledge co-creation. More specifically, we aim to further explore the phenomenon of knowledge co-creation in existing message boards for persons with disabilities by investigating community managers’ views on and experiences with knowledge co-creation.

In this study, we focus on pan-disability online communities and online communities for people with spinal cord injury in particular. Given the overarching consequences a spinal cord injury entails for those affected [54], we chose to explicitly include online communities for spinal cord injury in addition to pan-disability online communities, as they present a particularly interesting case in this context. Prior research suggests that people with disabilities increasingly use online and social media technologies such as message boards or mailing lists to find like-minded individuals to exchange their health-related experiences [55,56]. Under the biopsychosocial model of disability, these experiences are recognized as a valid form of expertise, originating from the person’s *lived experience* with a disability [57].

Our study differs from prior research with respect to two points. First, in addition to peer-to-peer knowledge co-creation, we also explore knowledge co-creation between community members and external actors, such as health care professionals, researchers, and businesses. Second, by focusing on the community managers’ perspective, we aim to gain a more in-depth understanding of the process of knowledge co-creation taking place in OHCs that is deeply rooted in the experiences of those users most familiar with the community and its members. Due to their role in the community, community managers possess insider-knowledge that extends beyond what is visible to regular community members and external observers. This study thus provides important insights on community managers’ views on knowledge co-creation in OHCs taking place between different actors, offering a new perspective on the interactions taking place in OHCs.

## Methods

### Study Design

As this study is explorative in nature, our aim was not to explain or proof but rather to provide a rich description of the phenomenon under investigation. We thus adopted a descriptive qualitative research design, following the consolidated criteria for reporting qualitative research guidelines (see Multimedia Appendix 1) [58]. According to Braun and Clarke, descriptive qualitative work aims to “‘give a voice’ to a topic or a group of people, particularly those we know little about” [59]. In line with this aim, the focus of our study was to give a voice to the community managers of existing online communities for people with disabilities to explore their views and experiences related to knowledge co-creation in their respective communities. Data were collected through semi-structured interviews with community managers. To complement the interview data, additional structural and administrative information was collected from the respective communities. To document and reflect upon the research process, a study journal was used, capturing the researchers’ underlying values and assumptions. The project was conducted in accordance with ethical guidelines for Web-based research proposed by Eysenbach [60].

### Participant Recruitment

A purposeful sampling approach was adopted. To identify English-language disability OHCs, we conducted a Google search with a combination of the terms “online community,” “disability,” and “spinal cord injury.” One author (JA) screened the first 100 search results, applying the following inclusion criteria: interactive, health condition-specific (disability), targeted at patients, and English as the main language. Both organization-initiated and individual-initiated OHCs were included. The search led to the identification of 22 OHCs that met the inclusion criteria: 12 spinal cord injury specific and ten for disability in general. A total of three OHCs were closed at the time they were identified. Personalized messages were sent to the indicated contact persons of all 22 platforms to inform them about the study and its purpose and to invite them to participate in a Web-based interview. Individuals were given the choice of an oral (video-calling) or written (email or chat) interview. Out of the 22 platform managers contacted, ten did not reply, two declined with no reason, and two confirmed interest but did not react to follow-up messages that were sent out three weeks after the initial invitation to participate.

### Data Collection

Data collection was carried out from October 2015 to June 2016. A total of nine semi-structured interviews (three email, five video-calling, and one face-to-face) were conducted with community managers of the remaining eight online communities (five spinal cord injury specific and three pan-disability). In one of the included OHCs, two moderators agreed to participate in the interview. All interviews were conducted by one author (JA), a female PhD student in health communication, trained in qualitative research methods with a particular research interest in patient participation. Informed consent was obtained from all participants. The semistructured interview guide was developed by the two authors and was not guided by a preexisting framework to allow for a certain degree of flexibility, enabling us to identify and follow up on participants’ individual experiences. The interview guide consisted of four subsections exploring participants’ perceptions and experiences with (1) the platform and its challenges, (2) open and user innovation, (3) external inquiries to interact with the community (eg, requests to participate in marketing research), and (4) members’ reactions to external inquiries (see Multimedia Appendix 2). Oral interviews lasted between 30 min and 1.5 h and were audio-recorded and transcribed verbatim. To complement the interview data, we retrieved additional information from the communities where available, including structural information (member lists, number and names of topic areas covered by the forum) and administrative information (terms and conditions and privacy statements, forum rules). Member lists were not available for some of the communities (C5, C7, and C8). Observations were documented in form of field notes by one researcher (JA). Once data collection was completed, informal exchange related to the study took place between one researcher (JA) and three community managers (M3, M4, and M5) via the private messaging function of the respective communities.

### Data Analysis

Data were analyzed using inductive thematic analysis following the six stages of coding and analysis proposed by Braun and Clarke [59], where the analysis is generated from the data (bottom-up) rather than shaped by existing theory. Here, however, Braun and Clarke note that the analysis is always to a certain degree shaped by the researcher’s standpoint and knowledge [59]. Two researchers, both health communication scholars, were involved in the analysis. The first researcher (JA) coded the majority of the material in an iterative process. A second researcher (SR) read and reflected on the material, providing an independent view on the data. We started by familiarizing ourselves with the data material. In the next step, the interview transcripts, terms and conditions and privacy statements, forum rules, and field notes were manually highlighted, coded, and collated. Upon this initial coding phase, recurring themes were identified in the material, and codes were collated into tentative themes. Our conceptualization of knowledge co-creation was primarily based on the coding of those examples drawn from the data, where interaction between members of the community was addressed that extended beyond emotional support and involved the creation of new ideas or knowledge. Data saturation was reached as indicated by the repetition in themes after the seventh interview. Regular meetings were held throughout the entire analysis process to reduce a potential bias. In case of a disagreement, we drew on the original data material and coding to reach a consensus.

## Results

### Reporting

To warrant the anonymity of the OHCs under investigation and in compliance with guidelines for conducting Web-based research proposed by Eysenbach [60], any information that would allow readers to draw inferences about the respective OHCs was omitted. In the text, interview quotes are attributed to the respective participant by using a participant identifier. In cases where forum content is quoted, compound quotes were used. The Results section is structured as follows. First, we provide some contextual findings in form of general information about the included communities. In the next step, we then present our findings related to community managers’ views on and experiences with knowledge co-creation in online communities for people with disabilities.

**Table 1:** Community characteristics.

Community	Community manager (role)	Focus	Size
Individual-initiated			
Community 1 (C1)	M1 (founder and moderator)	Spinal cord injury	Large
Community 2 (C2)	M2 (founder and moderator)	Spinal cord injury	Medium
Community 3 (C3)	M3 (founder and moderator)	Spinal cord injury	Small
Community 4 (C4)	M4 (founder and moderator)	Spinal cord injury	Medium
Community 5 (C5)	M5 (moderator)	Pan-disability	N/A^a^
Community 6 (C6)	M6a (moderator)	Pan-disability	Small
	M6b (moderator)		
Organization-initiated			
Community 7 (C7)	M7 (moderator)	Spinal cord injury	N/A^a^
Community 8 (C8)	M8 (moderator)	Pan-disability	N/A^a^

^a^N/A: not applicable.

### Characteristics of the Studied OHCs

Out of the eight communities investigated, five were initiated by individuals directly affected (C1, C2, C3, C4, and C5), one by an individual whose health status is unknown (C6), and two by organizations that involve volunteers who initiate and moderate forum discussions (C7 and C9). Out of the eight communities, three were classified as pan-disability (C5, C6, and C8), and five were focused on spinal cord injury (C1, C2, C3, C4, and C7). Communities were classified as large, medium, and small according to the number of registered members: small ones having less than 1000 members, medium-sized ones having between 1000 and 2500 members, and large ones having more than 2500 registered members. The study included both content moderators as well as community founders (Table 1). All of them indicated to have administrative rights to perform activities such as editing or removing content and blocking users, which is why from now on we refer to them as community managers. Table 1 presents an overview of the characteristics of the communities included in this study.

Similar to most message boards, each of the communities was divided into different sections and subsections, covering a wide array of topics related to disability such as *adaptive sports and recreation, assistive devices and technology, work life,* and *health issues.* With respect to access and openness of the respective communities, we found that most communities did not require users to register to view forum content, whereas some did have members-only sections for more personal topics such as relationships and sexuality. Only one of the communities was entirely members-only (C4). In all the communities, registration was required to actively share content on the forum, for example, posting a new thread or answering to an existing one. In addition to using the message board, registered users could also send private messages. Four of the communities entertained a chat room (C2, C3, C4, and C5), allowing for synchronous communication between users. Registration was not required to access and interact with other users in the chat rooms provided by two of these communities (C2 and C5).

### The Role of Community Managers

As founders or as assigned moderators, community managers have more power than regular community members. In their function as the community’s authority, they ensure that all members adhere to the community’s guidelines and rules. Not only can community managers edit, move, or pin content, but they can also permanently remove content from the community. The decision whether a contribution is in violation of the rules is entirely up to the community manager as reflected in the forum rules and terms and conditions statements of the communities investigated in this study (C1-C8). Community managers are the ones who make and enforce the rules, as clearly stated in the forum rules of the respective communities (C1-C8). Forum rules further state that community managers reserve the right to, at their sole discretion, modify or remove content. In addition, they also reserve the right to revoke membership to the forum and ban members from the community, temporarily or permanently, without prior notice or warning by blocking their Internet protocol address.

Community managers reported sometimes spending several hours a day taking care of the community, as one participant explained:

Whether I’m active on the site or not, I’m usually investing my time into finding information and stuff like that. (M6a)

Another participant mentioned he tries to “respond to every topic” (M3) he can, speaking of:

...hundreds of hours building [the community] and then thousands of hours managing it. (M3)

Two of the community managers (C1 and C8) reported being predominately involved in technical, strategic, and safeguarding issues, as one of them explained:

I do not tend to initiate discussions too much, as forum members start their own discussions. (M1)

Being in control of both the content and access to the community, community managers play an essential role in the knowledge co-creation process taking place in the respective communities. They act as the community’s boundary managers and gatekeepers who monitor all interaction and determine the interpretation of the rules and policies. In their role as the community’s authority, community managers can, therefore, facilitate or inhibit knowledge co-creation between members by removing or editing content, banning users, or by restricting access to certain areas of the community (eg, members-only areas).

### Knowledge Co-creation

After having provided some contextual information about the communities, we now present our findings related to community managers’ views on and experiences with knowledge co-creation in the communities under investigation. The analysis of the structural information extracted from the communities revealed that the number of registered users varied greatly between the communities, with some having less than 1000 registered users and others having several thousand users (see Table 1). Despite these discrepancies, all of the community managers reported having a small number of highly active users who are the main source of content, as summarized by one of the participants:

Active forums are usually maintained by moderators and a core membership who usually regularly post. (M6b)

These findings are supported by evidence gained from the structural information extracted from the communities. When comparing the number of replies to a post with the number of times it had been viewed, we found strong discrepancies, with much higher numbers in views than replies, indicating that users were much more likely to consume information than to actively post information (C1-C8). The comparatively small number of active users also became apparent when analyzing the communities’ member lists, which in addition to the username, usually also displayed information related to the users’ contribution behavior such as the number of posts and the number of likes.

Despite relatively small numbers of active contributors, community managers reported that knowledge co-creation did occur in their respective communities. These knowledge co-creation activities and community managers’ views on them constituted the main focus of our analysis. Our analysis revealed that there are different forms of knowledge co-creation in online communities for people with disabilities. More precisely, we identified two main themes: *peer-to-peer knowledge co-creation* and *types of collaboration with external actors,* including several subthemes (see Multimedia Appendix 3). In the context of this research, the terms *professional* or *external stakeholder* refer to health care professionals, researchers, students, and businesses alike, as we found that community managers rarely distinguished between the different types of external inquiries in their narrations. In the following subsections, we present a narrative account of our findings.

### Peer-to-Peer Knowledge Co-creation

In the peer-to-peer context, co-creation captures users’ joint efforts to develop new or modify existing products and services by actively building on each other’s ideas and insights. Our findings indicate that the idea of jointly creating new products that are more tailored to the actual needs of the disabled community appealed to the community managers. It was in fact an activity they strongly encouraged by asking members to share and exchange their experiences with products and services to develop new ideas, as outlined by one of the participants:

I mean that’s why we tell people to leave comments and ideas on the comments section below. To see if maybe, I don’t know, if they could come up with either a similar product that does a better job doing it, and they could share that with people, that would be cool. [...] I mean that’s what we want, its people to come up with ideas that generate innovation and new products and—to make our lives easier so we can regain some of the independence that we lost when we got injured. (M4)

In this context, one of the participants referred to his experiences outside the community, acknowledging the power and revolutionary nature of online communities in combination with advancing three-dimensional (3D) printing technologies. He particularly emphasized the driving force that online communities constitute in promoting the sharing of ideas and co-creation:

I’ve seen this very much in the 3D printing world recently—it has joined with the disabled world. [...] People are printing and making what’s the word, prosthesis orthosis, you know like splints, leg braces, and wrist braces—and they’ll be using 3D printers to do this. And it is revolutionary because usually these kind of things are crafted very meticulously by people in that line of industry. [...] And you know, this has been revolutionary and the sharing of information—nobody is trying to sell these blueprints or these 3D models, they’re all sharing the information openly. (M5)

The quote above illustrates how advancing technologies such as 3D printing, can empower and more importantly equip individuals with the tools they need to increase their independence and autonomy, reducing their dependence on professionals and experts who were traditionally the ones in charge of their health. It further highlights the altruistic aspects underlying co-creation in the peer-to-peer context and the idea of a “free flow of information” (M1) that allows individuals to use and build upon each other’s work to create new devices, tools, and ideas, as something beneficial within itself.

Despite community managers’ positive attitudes toward knowledge co-creation, we found that these activities were not yet common practice in the respective communities, where exchange involved users providing and receiving support rather than building on each other’s knowledge to create new knowledge and ideas, as illustrated by the quote below:

If someone would say, “I have got a problem putting a pair of trousers on,” then someone else would say “Well, I use, you know, this to do it.” or they got other techniques for doing it. And they help each other out. (M2)

So while participants recalled instances of users sharing ideas and making suggestions, there was a lack of concrete examples of knowledge co-creation efforts. When speaking of a section that was created to promote the exchange of users’ ideas and co-creation activities in a peer-to-peer format, the community manager of the respective community shared his experience, attributing the lack of interaction mainly to usability issues:

Unfortunately, so far there is not so much happening in this section [the one created to promote the exchange of users’ ideas]. I hoped that there would be much more but I think it’s also a problem of the usability of the website. (M7)

In addition, much of what related to co-creation, prototyping, and product development was still perceived to be controlled by professionals and experts. In this sense, most of the community managers mentioned how sharing ideas could inform product development, positioning themselves as informants to this process rather than claiming a more active role and decision power in the development and production phase:

We do get in ideas, disability aids mostly, good wheelchairs, what people want from them. But really it’s mostly all suggestions at the moment and not many people are coming together to make new ideas. [...] It’s just a case of how to implement it, to make people feel like they can have an input [...] I think if we’d have companies post on the site, say “We are interested in what you think” then more people would look into it. (M6a)

The quote above shows the perceived dependence on manufacturers, suggesting that community members’ ideas were regarded as input that could only realize its value once it was taken up by professionals (eg, manufacturers). It suggested that community members do not see a purpose in sharing their ideas if there is no business interested in producing them.

### Types of Collaboration With External Actors

All of the communities allowed members of different groups, including researchers, health care professionals, and students to join and were open to collaborating with them, as long as their involvement was not purely commercially motivated. Any form of pure advertisement was strictly forbidden, as outlined in the forum rules and terms and condition statements of the respective communities (C1-C8). One of the participants summarized:

We allow members with different disabilities, service providers, and charities to join discussions if it’s not simply for commercial gain. [...]. We are in favor of assisting with research participation where able. [...] We also allow requests on the forum regarding product research and development, however research for the sole purpose of profiting from members opinions is discouraged. (M1)

Despite low numbers of businesses actively seeking the communities’ insights, community managers were also open to them joining their respective communities as long as they “are transparent as to who they are” (M6b) and “willing to get involved in the conversations and do not just use the forum to promote their products or services” (M6b). Community managers, in fact, underlined the benefits and importance of businesses looking at the ideas and insights users are sharing online and “to listen to disabled consumers” (M8). In these statements, participants expressed hope that this may help to improve the life of those living with a disability by creating a better understanding of what their needs are.

According to the participants, most of the external inquiries related to research projects of students or health care professionals in training. In this context, it was noted that collaboration did usually not take place in an interactive manner or over longer periods. Most of the time, there was a set of questions that community members were asked to answer, for example, in the form of a poll or a survey. According to the community managers, researchers and students often joined the community for the sole purpose of recruiting participants for scientific studies and usually did not have the intention of getting involved with the community or to follow up, for example, by sharing or discussing their research findings with the community members. In other words, they recognized that these forms of exchange were unilateral, limited in time, and in pursuit of a clear goal set by the person seeking the community’s insights. As one participant explained:

Usually, it’s just you know “I’m studying something would you mind if I asked a few questions?” And nobody would ask to see the results or to read the paper—and that’s usually it, that’s the extent of our interaction. [...] You know they come, they ask the questions, they leave. You know, they just use us. Well, because they need to continue their own path, you know their education. They were always very clear that they were doing a study and were looking for volunteers [...] it was always clear what the purpose was. (M5)

The notion of “they use us” in this context reflects frustration regarding the unilateral nature of the collaboration and the lack of true involvement with the community and its needs. Comments as the one above, however, also show that community managers understand and accept the reasons for this form of interaction from the perspective of nondisabled researchers, health care professionals, and students. For them, the mere interest of these user groups in their community was already perceived as beneficial within itself, recognizing it as an important first step. In this sense, most of the community managers underlined the importance of welcoming students, researchers, health care professionals, and businesses to spread important information and help to inform health care research and practice. As one of the community managers commented:

Educating people on this matter is good, can’t really harm us at all—it’s a good thing, you know, it’s good to raise awareness. (M3)

Another participant described it as a win-win situation, turning the traditional patient education approach around, highlighting the community’s role in educating professionals, helping them to gain a better understanding of persons with disabilities and their needs. He explained:

We’re educating tomorrow’s doctors, we’re educating tomorrow’s nurses, tomorrow’s engineers in some cases. [...] We’ve all had bad experiences with doctors or nurses, physiotherapist or occupational therapists—so the general idea is: The more we can help them, the better they will be. (M5)

As illustrated by the quote above, being able to contribute to advancing research and improving practice was an essential aspect voiced by the part(icipants. The underlying hope expressed by participants in this context is not only to help oneself but rather to also “improve stuff for everyone else [living with a disability]” (M2), including not only products but also treatment. In this context, participants emphasized the importance of knowledge dissemination, describing it as a “ripple effect” by which good ideas are spread (M2). One participant particularly emphasized the community’s readiness to take on a more active role in the health care process:

I just want the information to be out there. That I’m not just another sick person sitting in my room 24 hours a day, you know. I just want to let the world know that we are people—we are not just disabled. At the back of the community we have brains and we want to use them. (M6a)

Besides the generally positive attitudes of community managers, some of them recalled instances where community members expressed skepticism toward external inquiries, as one of them explained:

Feedback from users is that they felt like they were just being used for free research, so we aim to keep these [external inquiries] separate for other conversations. (M8)

Comments as this one reflect the frustration experienced by certain users who felt exploited by external requests, which were often time-consuming and did not offer any immediate benefit to users. These users feel disturbed, perceiving external inquiries as an intrusion to their privacy and personal space. Unlike community managers, they are focused on their own situation and do not always see the big picture. In this context, one of the community managers recalled the need to introduce a new policy that clearly stated that students and researchers were welcome in the community to counterbalance users’ expression of “negative attitudes like ‘Oh no, I’m not nobody’s Guinea pig, I don’t want to be, I get enough questions asked from doctors!’” (M5)

In this context, community managers underlined how the adoption of a give-and-take approach by researchers could make a difference in users’ perceptions and how this would in turn help to establish trust and encourage co-creation. Even though some researchers offered vouchers or gift certificates as compensation for members’ time and effort to participate in a study, we found that actions such as “making research fun” (M4), avoiding lengthy questionnaires, and sharing research findings with the community were perceived as equally important by community managers:

When people agree to [sharing their research findings], I guess you feel a little less used because you’ve seen the result. You’ve seen how it has helped somebody and you see the light in which their information has been used. (M5)

In the context of an increasing number of external inquiries, some community managers have emphasized the need to shield the community and its members from too many external inquiries, as they may disrupt the communication taking place between regular users. So to protect the community members’ interests, they established clear rules such as “dedicated areas for research requests” (M1) and other external inquiries, separating these requests from the general discussion taking place in the community. This separation serves a similar purpose as the separation of editorial and advertising content in mass media—protecting consumers by ensuring transparency and editorial integrity. An additional precaution taken by community managers was to review external inquiries to ensure that they adhere to the rules and regulations of the respective communities. In this context, community managers reported checking whether requests originated from a legitimate source and whether they complied with “professional standards.” (M6b)

There was only one community (C7) that did not allow any external inquiries to be posted to the community, acknowledging that measures taken against these inquiries, such as removing posts by students seeking to recruit study participants, were not only in the best interest of the community members but also motivated by the organization’s own agenda:

We also want to do studies in the future with users so if they get one call every day, the motivation to participate might decrease a lot. So we also want to check that they don’t get too many offers for study participation. (M7)

## Discussion

By adopting the community managers’ perspective, our results reflect the experiences, views, and in-depth knowledge of those members who play a key role in governing their respective communities. Thus, our results offer a unique insider perspective on what is happening *behind the scenes* of the included OHCs beyond what is publicly visible. In the following paragraphs, we critically discuss our findings and provide recommendations on how these findings can be leveraged to foster knowledge co-creation in online communities.

### Principal Findings

Our findings contribute to existing research in that they highlight the currently under-investigated role of OHCs as platforms for collaboration and co-creation between patients, health care professionals, businesses, and researchers. By taking online communities for people with disabilities as a case in point, we aimed to highlight the potential of existing OHCs to contribute to the improvement of products, services, and research.

In this paper, we explored community managers’ views and experiences in relation to knowledge co-creation in online communities for people with disabilities. Here, we identified two main themes: *peer-to-peer knowledge co-creation* and *types of collaboration with external actors.* On the one hand, our findings showed that most community managers had positive attitudes toward knowledge co-creation. Here, they highlighted the potential of knowledge co-creation to improve health care service delivery as well as its positive impact on individual care situations. They also advocated for openness and a free flow of information to promote co-creation among patients and between patients and professionals. On the other hand, community managers also stressed the need to establish and enforce certain ground rules for collaboration to protect the community’s interests, particularly with respect to the involvement of external stakeholders. Although we identified positive attitudes toward knowledge co-creation and examples of collaborative efforts involving the exchange of information, concrete examples of knowledge co-creation were scarce, indicating a lack of concrete experiences community managers could refer to. This, in turn, suggests that whereas community managers are not only open but supportive of knowledge co-creation, it is not yet taking place to the extent they would hope for. As a result, much of the knowledge that resides within the OHCs under investigation lies idle.

Whereas prior research has shown that patients can come up with innovative ideas and solutions [23,61,62], less is known about whether and how these ideas can be captured and further developed in collaboration with other patients or health care organizations. In line with previous research in the marketing and management literature [53], we found that existing OHCs constitute a promising way of fostering knowledge co-creation and innovation. Indeed, our findings suggest that community managers have positive attitudes toward knowledge co-creation, providing a fruitful and supportive environment for these activities to take place. Here, participants indicated an interest to contribute not only as participants but as collaborators, taking on tasks such as assisting in the formulation of relevant research questions, assisting with data collection, prototype testing, or product reviewing, acknowledging that in this way a much wider patient population could benefit. In fact, community managers promoted and encouraged their communities to be active in sharing their experiences not only to help others but also to create new knowledge to educate health care professionals and to help advance research. This active role described and promoted by community managers is also a key issue addressed by a paradigm shift in disability studies, most well known for its mantra “Nothing About Us Without Us” [63]. Here, many patient advocates, as well as scholars, have argued that research should embrace the experiential knowledge of persons with disabilities. It has further been highlighted that participatory research, which builds from socially informed models of disability, constitutes an approach benefiting both individuals as well as the quality of the research [64]. Even though participatory research is gaining increasing attention, particularly in the field of disability studies, it is not clear how persons with disabilities should be identified as collaborators in these projects [65]. Findings of our study indicate that whereas online communities for people with disabilities are interested in collaborating with researchers and practitioners, they are currently not involved in this process. In the following paragraphs, we outline why we believe OHCs constitute a promising way of fostering knowledge co-creation between different stakeholders in the disability context.

Whereas in health care we usually aim for representativeness, we here draw on lead user theory to make a case for focusing on knowledge co-creation with those individuals who we refer to as lead users. Lead user theory [66] describes lead users as those users of a certain product or service who are (1) early adopters of the product or service, (2) ahead of an important market trend, and (3) experiencing high benefits from innovating. Lead users usually experience needs before the general market does, and in the absence of adequate solutions, they innovate to fulfill their needs. According to lead user theory, this makes them a promising source of innovative ideas to generate new products and services. Indeed, there is a growing body of literature on open and user innovation, providing strong empirical evidence that lead users are likely to come up with commercially lucrative innovations [66-68]. To harness these innovations, it has been suggested to integrate lead users into the corporate innovation process using the lead user method [66,67,69]. The lead user method, as proposed by von Hippel [66], enables companies to identify and capture both lead users’ needs as well as their ideas and solutions, allowing them to derive promising ideas for new products and services.

Applying the concept of lead users to the health care context, it was suggested that disabled persons adhere to two key attributes of lead users originally defined by von Hippel [65]. Rather than considering persons with a disability as lead users merely as a result of their disability, we propose that in the case of persons with disability, it is also individuals’ high product or service use experience that can be a driver for innovative ideas and motivation to engage in knowledge co-creation [70]. Moreover, we build on prior lead user research conducted in online communities of practice, which suggests that lead users are highly likely to be able to provide knowledge to the community and also do so, given the low cost of providing knowledge they have readily available. Indeed findings show that lead user characteristics relate positively to making contributions to the community [71].

Interpreting our findings in light of these considerations, we argue that community managers, as well as other active core members who actively contribute to OHCs, are likely to possess lead user attributes, making them an important resource of innovative ideas for health care organizations and researchers. Hence, we propose that existing OHCs can help researchers and practitioners to identify and get in touch with lead users who, as our study has shown, usually constitute a small core community, with community managers acting as gatekeepers. We further suggest that existing OHCs can also serve as a platform for knowledge co-creation. Here one of the key benefits is that knowledge co-creation can take place independent of time and geographical restrictions, as it does not require individuals to meet face-to-face. In this way, it may also help to include individuals who may not be able to participate in traditional face-to-face focus groups or interviews because of reduced mobility.

### Practical Implications

Previous research has shown that OHCs can not only be an important resource for patients and their families but also for health care professionals and researchers [11,21]. However, as outlined earlier, building and maintaining such platforms constitutes a resource-intensive endeavor without guaranteed success [9,52]. In this study, we found that there are several active online communities for people with disabilities that are very much interested in and open to collaborating with different stakeholders such as health care professionals, researchers, students, and businesses to create ideas and new knowledge. These findings are in line with previous research [15] and emphasize the need to harness existing resources to realize the potential of fostering relationships between researchers and patients via OHCs.

We thus propose that collaborating with existing OHCs may, in fact, be a promising alternative to setting up entirely new communities, as it reduces efforts related to attracting and maintaining community members. This, in turn, allows also those institutions or individual professionals who may lack the needed resources to build and maintain an active community themselves to engage with well-established OHCs. However, gaining access to these communities can be challenging [72-74]. On the basis of our findings and in line with ethical recommendations for conducting health research online [60], we propose that the most efficient way of gaining access to a community is through the community manager. In their role as gatekeepers, community managers are in control of content and access to their respective OHCs and thus play an essential role in the knowledge co-creation process [75]. In this context, particular attention should be paid to the considerable impact that involving gatekeepers may have not only on the quantity and quality of data collected [76] but also on the research project as a whole [74,77]. Community managers may, for example, influence how a particular research project is presented to the community. This framing of a project may in turn influence not only how the project is understood by community members but may also influence their response and participation behavior.

However, even though existing OHCs provide a promising platform to promote knowledge co-creation between patients, health care professionals, researchers, and businesses, there are some important aspects to be considered. First, there are considerable challenges related to the adoption of eHealth initiatives on the part of professionals who are concerned about the additional benefits of Web-based tools, the effort needed to implement and sustain them, as well as issues relating to workload, role clarity, and accountability [17,78,79]. It will thus be essential to provide clear evidence and guidelines on how OHCs can be used to facilitate knowledge co-creation in health care and how these activities can ultimately benefit each stakeholder group. Moreover, it is important to acknowledge that there are parts of the population lacking access, skills, confidence, or interest in using online communities [80-83]. Stakeholders should thus be attentive and, if possible, mitigate negative effects, for example, by combining co-creation activities taking place in OHCs with more conventional face-to-face approaches such as focus groups [13].

### Limitations and Directions for Future Research

Our study has some limitations, which are inherent to the qualitative research design we adopted. Recognizing that as researchers we cannot completely separate our beliefs and expectations from the subject of research [84], we tried to mitigate this potential bias through regular meetings throughout the course of the study. These meetings helped us to discern our own perceptions, allowing us to better understand and interpret our data to represent our participants’ experiences [84]. Given its exploratory nature and its focus on OHCs for people with disabilities, our findings are not generalizable. Furthermore, our results might be biased in that the included communities and community managers might be more open to collaboration and co-creation than those communities who declined to participate in the study or did not respond to our inquiry. Also, in our study, we did not include OHCs that were moderated by health professionals.

In light of the findings and limitations of this study, future research should further investigate knowledge co-creation taking place in different health condition–specific OHCs to gain a better understanding of the factors favoring and hindering knowledge co-creation and to identify best practice approaches. This may in turn also help to determine promising and less promising areas for investigation. In addition, it will be essential to demonstrate how OHCs can not only help to identify unmet patient needs but can also uncover ideas, tips, and tricks developed by patients themselves. These may be in the form of homemade assistive devices, innovative self-management techniques, or out-of-the-box thinking when it comes to interpreting research findings. In this context, it will be particularly important to compare and contrast online co-creation activities with traditional approaches to patient participation, such as face-to-face focus groups, to determine the true added value online communities have to offer.

In light of community managers’ essential role in the community, we recommend involving them not only at the stage of data collection, as it is currently common practice, but rather to collaborate throughout the entire research process to benefit from their in-depth knowledge of the community and its members. In addition to traditional dissemination strategies, we strongly recommend disseminating and discussing research findings with the communities involved in the project. A closer involvement of online communities in health care may indeed contribute to fostering knowledge dissemination, thus favoring knowledge translation [85,86]. As such, it may be beneficial for patients and health care professionals alike [87]. In this context, it could be particularly interesting to also further investigate online interactions related to co-creation taking place between patients and health professionals who act as moderators of OHCs [87].

### Conclusions

This paper enriches our understanding of OHCs by providing a rich description of community managers’ views on knowledge co-creation in online communities for people with disabilities. Findings of our study indicate that whereas online communities for people with disabilities are interested in collaborating with researchers and practitioners to create new ideas and knowledge, they are currently not involved in this process. By building on lead user research, we draw attention to the currently under-investigated role of online communities in fostering knowledge co-creation between different stakeholders in the disability context. In doing so, we suggest that innovative ideas may not necessarily emerge from traditionally used forms of health care research focused on covering a representative sample of individuals. Rather we propose that they may result from engaging lead users, who possess the required skill, knowledge, and motivation to engage in knowledge co-creation and are likely to come up with innovative ideas on how to modify and improve existing health care services, products, and research.

Here, we argue that community managers, as well as other core members who actively contribute to online communities, are likely to possess lead user attributes, making them an important resource of innovative ideas for health care organizations and researchers. We thus believe that existing online communities can help researcher and practitioners not only to identify lead users but that they can also serve as a platform to foster knowledge co-creation between patients, health care professionals, researchers, and businesses. Ultimately, knowledge co-creation will help to inform the development of products, services, and research activities that better meet the needs of those living with a disability. This study provides some initial insights into knowledge co-creation in online communities for people with disabilities; however, more research is needed to better understand and harness this new role of OHCs.

## Appendix

Multimedia Appendix 1 Consolidated criteria for reporting qualitative research checklist.

Multimedia Appendix 2 Interview guide.

Multimedia Appendix 3 Overview of themes and subthemes generated from the analysis.

## Abbreviations

OHC: online health communities
3D: three-dimensional
