# Supplementary material for: Views of Community Managers on Knowledge Co-creation in Online Communities for People With Disabilities: Qualitative Study
Source: J Med Internet Res. 2017 Oct 10;19(10):e320. doi: 10.2196/jmir.7406 (PMC5654737; doi:10.2196/jmir.7406)
Supplement: Multimedia Appendix 1 [file jmir_v19i10e320_app1.pdf]

Consolidated criteria for reporting qualitative studies (COREQ)

No.	Item	Guide questions/description	Reported on page
**Domain 1: Research team and reflexivity**			
**Personal Characteristics**			
1	Interviewer/facilitator	Which author/s conducted the interview or focus group?	10
2	Credentials	What were the researcher's credentials?	10
3	Occupation	What was their occupation at the time of the study?	10
4	Gender	Was the researcher male or female?	10
5	Experience and training	What experience or training did the researcher have?	10
**Relationship with participants**			
6	Relationship established	Was a relationship established prior to study commencement?	9-10
7	Participant knowledge of the interviewer	What did the participants know about the researcher?	N/A
8	Interviewer characteristics	What characteristics were reported about the interviewer/facilitator?	10
**Domain 2: study design**			
**Theoretical framework**			
9	Methodological orientation and Theory	What methodological orientation was stated to underpin the study?	4-11
**Participant selection**			
10	Sampling	How were participants selected?	9
11	Method of approach	How were participants approached?	9-10
12	Sample size	How many participants were in the study?	10
13	Non-participation	How many people refused to participate or dropped out? Reasons?	10
**Setting**			
14	Setting of data collection	Where was the data collected?	10
15	Presence of non-participants	Was anyone else present besides the participants and researchers?	10
16	Description of sample	What are the important characteristics of the sample?	11-12
**Data collection**			
17	Interview guide	Were questions, prompts, guides provided by the authors? Was it pilot tested?	10
18	Repeat interviews	Were repeat interviews carried out? If yes, how many?	N/A
19	Audio/visual recording	Did the research use audio or visual recording to collect the data?	10
20	Field notes	Were field notes made during and/or after the interview or focus group?	10
21	Duration	What was the duration of the interviews or focus group?	10
22	Data saturation	Was data saturation discussed?	11

**23**	Transcripts returned	Were transcripts returned to participants for comment and/or correction?	N/A
**Domain 3: analysis and findings**			
**Data analysis**			
**24**	Number of data coders	How many data coders coded the data?	11
**25**	Description of the coding tree	Did authors provide a description of the coding tree?	N/A
**26**	Derivation of themes	Were themes identified in advance or derived from the data?	11
**27**	Software	What software, if applicable, was used to manage the data?	N/A
**28**	Participant checking	Did participants provide feedback on the findings?	10
**Reporting**			
**29**	Quotations presented	Were participant quotations presented to illustrate the themes / findings? Was each quotation identified?	11-20
**30**	Data and findings consistent	Was there consistency between the data presented and the findings?	10-20
**31**	Clarity of major themes	Were major themes clearly presented in the findings?	10-20
**32**	Clarity of minor themes	Is there a description of diverse cases or discussion of minor themes?	19-20
