# Supplementary material for: Views of Community Managers on Knowledge Co-creation in Online Communities for People With Disabilities: Qualitative Study
Source: J Med Internet Res. 2017 Oct 10;19(10):e320. doi: 10.2196/jmir.7406 (PMC5654737; doi:10.2196/jmir.7406)
Supplement: Multimedia Appendix 2 [file jmir_v19i10e320_app2.pdf]

Interview Guide

The platform and its challenges

1. Why did you decide to start your own online community? (*only for founders*)
   What is your role in the community?
2. What distinguishes your community [NAME] from other online communities for people with disabilities? What makes it popular?
3. Where do you see the challenges in
   - attracting members?
   - maintaining an active user community?
4. Do you invest a lot of time in moderating and initiating discussions?

Open innovation communities in the field of healthcare

5. Have you ever heard of user innovation communities before?
   - Yes: What have you heard and what do you think about them?
   - No: Interviewer explains and provides examples
6. Do you think that user innovation communities could work in the healthcare setting?
7. Do you consider your community to be an open innovation community?
8. Do you see a potential for knowledge co-creation[1] in your community?
   - What may be the challenges or risks?
   - What may be the benefits?
9. What about non-members, do they benefit from the information? Does information that's shared within the online community "leave" the online world?
   - Is it taken up by patients or healthcare providers?
   - Why or why not? Do you have any examples?

External Inquiries

10. Have you been contacted by other researchers or healthcare professionals before?
11. Have you been contacted by businesses before that wanted to use your community for marketing research?
12. Does it happen that external actors do not obtain your permission before interacting with the community?
    - How do you find out about these cases and how do you deal with them?
13. Are you worried about external actors "stealing" ideas from the community and making profitable products/services out of them?

Community reaction to external inquiries

14. How do the community members react to inquiries from external actors?
    - Can you think of a specific example?
15. How are calls for participants for research projects perceived by the community?
    - Why do you think this is the case? Do you have an example in mind?
16. Are users willing to collaborate with firms for product and service development?
    - Why do you think this is the case? Do you have an example in mind?

---

[1] Where the concept of co-creation was unclear it was explained using the example of a well-known children's toys manufacturer who actively involves an online community in knowledge co-creation
