# Supplementary figures and images for: Views of Community Managers on Knowledge Co-creation in Online Communities for People With Disabilities: Qualitative Study
Source: J Med Internet Res. 2017 Oct 10;19(10):e320. doi: 10.2196/jmir.7406 (PMC5654737; doi:10.2196/jmir.7406)

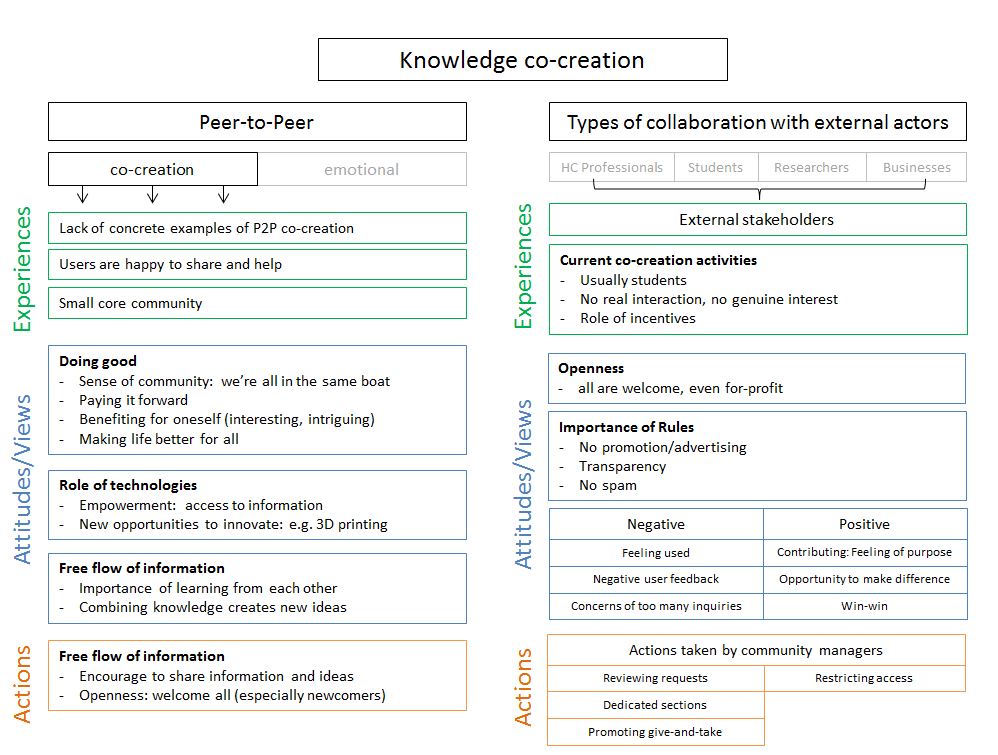

Supplement: Multimedia Appendix 3 [file jmir_v19i10e320_app3.JPG]
